# Expression of Concern: Induction of apoptosis in rat lymphocytes by starvation

**DOI:** 10.1042/CS20060212_EOC

**Published:** 2026-08-25

**Authors:** Juliana Pires, Rui Curi, Rosemari Otton

**Affiliations:** 1 Department of Physiology and Biophysics, Institute of Biomedical Sciences, University of São Paulo, Av. Prof. Lineu Prestes, 1524, Butantan 05508-900, São Paulo, SP, Brazil; 2 Cruzeiro do Sul University, Biological Sciences and Health Centre, Regente Feijo, 1295, Analia Franco 03342-000, São Paulo, SP, Brazil

**Keywords:** apoptosis, cell death, cytochrome c, fatty acid, immune function, lymphocyte, starvation

*Clinical Science* has been made aware of potential issues within this paper and is hence issuing a permanent Expression of Concern to notify readers.

A reader identified a region of high similarity within both the Figure 4A and 4B panels, and upon investigation, it was identified that the Figure 2 gel showed evidence of lane rearrangement. The authors provided an explanation and raw data, including coloured images for the relevant Figure 4 panels; however, the control image showed a shifted field of view and not all images correlated with the Figure panels with certainty in all points. The full gel was provided for Figure 2, which included all the lanes in the Figure as well as additional lanes, which were removed to create the Figure; this rearrangement was not described in the legend.

The authors fully cooperated, but some ambiguities remain. As such, the article will remain published but with an associated Expression of Concern. The authors confirm that the reported Results and Conclusion remain unaffected.

